# Estrogen receptor alpha gene polymorphism and endometrial cancer risk – a case-control study

**DOI:** 10.1186/1471-2407-8-322

**Published:** 2008-11-06

**Authors:** Sara Wedrén, Lovisa Lovmar, Keith Humphreys, Cecilia Magnusson, Håkan Melhus, Ann-Christine Syvänen, Andreas Kindmark, Ulf Landegren, Maria Lagerström Fermér, Fredrik Stiger, Ingemar Persson, John A Baron, Elisabete Weiderpass

**Affiliations:** 1 Institute of Environmental Medicine, Karolinska Institutet, Stockholm, Sweden; 2 Department of Medical Sciences, Uppsala University, Uppsala, Sweden; 3 Department of Medical Epidemiology and Biostatistics, Karolinska Institutet, Stockholm, Sweden; 4 Department of Public Health, Karolinska Institutet, Stockholm, Sweden; 5 Department of Genetics and Pathology, Uppsala University, Uppsala, Sweden; 6 AstraZeneca R&D, Mölndal, Sweden; 7 Swedish Medical Products Agency, Uppsala, Sweden; 8 Dartmouth Medical School, Hanover, New Hampshire, USA; 9 Department of Etiological Research, Cancer Registry of Norway, Oslo, Norway; 10 Department of Community Medicine, University of Tromsö, Norway; 11 Department of Genetic Epidemiology, Samfundet Folkhälsan, Helsinki, Finland

## Abstract

**Background:**

Estrogen is an established endometrial carcinogen. One of the most important mediators of estrogenic action is the estrogen receptor alpha. We have investigated whether polymorphic variation in the estrogen receptor alpha gene (*ESR1*) is associated with endometrial cancer risk.

**Methods:**

In 702 cases with invasive endometrial cancer and 1563 controls, we genotyped five markers in *ESR1 *and used logistic regression models to estimate odds ratios (OR) and 95 percent confidence intervals (CI).

**Results:**

We found an association between rs2234670, rs2234693, as well as rs9340799, markers in strong linkage disequilibrium (LD), and endometrial cancer risk. The association with rs9340799 was the strongest, OR 0.75 (CI 0.60–0.93) for heterozygous and OR 0.53 (CI 0.37–0.77) for homozygous rare compared to those homozygous for the most common allele. Haplotype models did not fit better to the data than single marker models.

**Conclusion:**

We found that intronic variation in *ESR1 *was associated with endometrial cancer risk.

## Background

Estrogens, whether from endogenous or exogenous sources, are potent mitogens in the endometrium and thus constitute a major carcinogen in this tissue [1]. Endometrial cancer is therefore a good model for investigating clinical effects of estrogen signaling. Estrogen receptor alpha is the principal estrogen receptor expressed in the endometrium [2,3] and it is considered to be crucial in the development of endometroid endometrial carcinoma, the most common histological subtype among endometrial neoplasms [1]. Since altered ER function could have impact on the risk of endometrial cancer, the estrogen receptor alpha gene (*ESR1*) is a plausible endometrial cancer candidate gene which has been investigated in a few studies [4-6]. We have investigated whether polymorphic variation in the *ESR1 *is associated with invasive endometrial cancer risk, overall and in subgroups defined by other hormonally related factors.

## Methods

### Parent study

This nation-wide population-based case-control study encompassed all cases of incident, primary histopathologically confirmed invasive endometrial cancer among women 50 to 74 years of age resident in Sweden between January 1, 1994 and December 31, 1995 as previously described in detail [7]. Endometrial cancer patients were identified at diagnosis through a notification system organized within the six Swedish regional cancer registries, to which reporting of all malignant tumors is mandatory. Controls were women randomly selected in 5-year age strata (to match the expected age frequency distribution among the cases) from the Swedish Total Population Register. Only women with an intact uterus and no previous history of endometrial cancer were eligible for this study.

Endometrial cancer patients were asked to participate by their respective physicians. After their informed consent, we mailed self-administered questionnaires asking for detailed information about use of menopausal hormones and oral contraceptives, weight, height, reproductive history, medical history, and other lifestyle factors. Controls were contacted directly with the questionnaire. Seventy-six percent of eligible cases (n = 802) and 84 percent of the controls (n = 3 550) ultimately participated in the parent study by answering the questionnaire. Among these controls, 491 who failed to return the mailed questionnaire were interviewed by phone. Slides with tumor tissue were collected from all cases and reviewed by one study pathologist to confirm the diagnosis, and to document tumor grade and myometrial invasion. The specimens were reclassified as: endometroid adenocarcinoma (n = 648), seropapillary carcinoma (n = 36), clear cell carcinoma (n = 10), adenoacanthoma (n = 3), adenosquamous carcinoma (n = 12), endometrial atypical hyperplasia (n = 80) (adenomatous hyperplasia with slight, moderate or severely pronounced atypia), and anaplastic carcinoma (n = 13). Results from the parent study have been published [7-10].

### Selection of present study population

In the analysis, we included all women with endometrial cancer (n = 802) and 802 randomly selected controls (frequency-matched by age) from the parent study. In order to increase statistical power in subgroup analyses, we additionally selected all remaining eligible controls who had taken menopausal hormone treatment, either preparations containing medium potency estrogen only (mainly estradiol or conjugated estrogens) or medium potency estrogen in combination with progestin for at least 2 years (277 controls) and all remaining control women with self-reported diabetes mellitus (124 controls). For a detailed description of the hormone treatment variables, please see [8]. In total, 802 cases and 1 203 controls were selected. In addition, we included 871 controls from the parent study who had also participated and been genotyped in a parallel breast cancer study, who fulfilled the inclusion criteria (including having an intact uterus). The Institutional Review Board at Karolinska Institutet, Stockholm, Sweden and the six other Swedish regional review boards approved this study.

### Collection of biological material

All selected living women were contacted by mail and those who gave informed consent received a blood sampling kit by mail. Whole blood samples were drawn at a primary health care facility close to the woman's home and were sent to us by standard mail. A majority of the samples arrived at Karolinska Institutet within 24 hours after blood draw. All blood samples were immediately stored at -20°C. Endometrial cancer cases who declined to donate a blood sample were asked to permit to our use of normal tissue from archived paraffin embedded tissue taken at cancer surgery (consisting of, for example, cancer-free lymph nodes, uterine tube or myometrium). We also attempted to retrieve archived tissue samples from all deceased endometrial cancer cases. Samples were coded and transferred to the laboratories. Blood samples or archived tissue samples were obtained for 603 and 104 endometrial cancer patients, respectively, and blood samples from in total 1 574 control women (922 selected for this study + 652 genotyped in the breast cancer study), yielding participation rates of 88 percent for cases (707 of 802 eligible) and 76 percent for controls (1 574 of 2 074 eligible). Considering also the participation rate in the parent study, overall participation rates were 67 percent and 64 percent among cases and controls, respectively.

### Selection of SNPs and microsatellite markers

We selected *ESR1 *polymorphisms to be analyzed from the literature [11-14]. The polymorphisms were not intended to capture variation across the entire gene. The 5' promoter microsatellite (TA_n_) rs2234670 is located -1174-base pairs upstream of exon 1, while the SNPs rs2234693 (also known as PvuII) and rs9340799 (also known as XbaI) are found in intron 1, and the synonymous coding rs4986934 (codon 243 CGC>CGT) and rs1801132 SNPs (codon 325 CCC>CCG) are located in exon 3 and 4, respectively.

### DNA extraction

DNA was isolated from 3 ml whole blood using the Wizard Genomic DNA Purification Kit (Promega, Madison, WI, USA) according to the manufacturer's instructions. From non-malignant cells in paraffin embedded tissue, we extracted DNA using a standard phenol/chloroform/isoamylalcohol protocol [15].

### Genotyping methods

#### SNPs and microsatellite markers

All primers and probes for the 5' promoter microsatellite rs2234670, the intron 1 SNPs rs2234693 and rs9340799, and the synonymous exonic rs4986934 and rs1801132 SNPs, were designed based on the reference sequences AF082876, AF326912 and NM_000125, respectively. For the fluorescence polarization and the minisequencing assays [16], we designed minisequencing primers complementary to the sequence immediately adjacent to the SNPs. For the Molecular Beacon assay [17], we designed two fluorescently labeled allele specific probes for each SNP, carrying the variable position in the middle of the loop region [18]. We used a web-based DNA folding program (mfold) [19] to estimate the stability of the stem and loop structure of the MB probes. Supplementary tables 1 and 2 (Additional file 1) give the primer and probe sequences and their modifications.

#### Minisequencing assay using fluorescence polarization detection

The region containing the intron 1 SNPs, rs2234693 and rs9340799, was amplified in one PCR fragment. PCR was performed using 20 ng of genomic DNA, 0.02 U/μl Taq Gold DNA polymerase (Applied Biosystems, Foster City, CA, USA), 100 μM dNTPs (Amersham Pharmacia Biotech, Uppsala, Sweden) and 0.1 μM of both PCR primers in 30 μl of GeneAmp PCR buffer supplied with the enzyme at 95°C for 7 minutes, followed by 40 cycles at 92°C for 15 seconds, 56°C for 1 minute and 72°C for 45 seconds. Five microliters of PCR product was aliquoted to 384-well microtiter plates and 5 μl of a PCR cleanup mixture containing 0.1 U/μl shrimp alkaline phosphatase (USB Corporation, Cleveland, OH) and 0.1 U/μl Exonuclease I (USB Corporation) in 20 mM Tris-HCl pH 8.0 and 10 mM MgCl_2 _was added.

The plate was incubated at 37°C for 1 hour, followed by 15 minutes at 95°C to inactivate the enzymes. Five microliters of extension mixture containing 5 μM extension primers, and 0.08 U/μl Thermosequenase (Amersham Pharmacia Biotech) in 50 mM Tris-HCl pH 9, 50 μM KCl, 5 mM NaCl, 5 mM MgCl_2_, 8% glycerol, and fluorescently labeled and unlabeled ddNTPs was added to each reaction. The two ddNTPs relevant for the particular SNP were labeled and included at a 1:5 ratio relative to unlabeled ddNTPs. The total concentration of ddNTP was 0.125 μM. The fluorescent dye rhodamine 110 (R110) was used to label ddGTP and ddCTP, and 6-carboxytetramethylrhodamine (TAMRA) were used to label ddATP and ddUTP (PerkinElmer Life Sciences, Boston, MA, USA). The cyclic extension reaction was performed at 92°C for 1 minute followed by 40 cycles of 92°C for 10 seconds and 55° for 30 seconds. The fluorescence signals in the 384 well plates were read on an Analyst AD™ (Molecular Devices Corporation, Sunnyvale, CA, USA). Genotypes were assigned using the software AlleleCaller ™ supplied with the instrument, and a custom-made Excel macro. In each run, positive controls for the three genotypes and negative controls were included. Both DNA polarities were analyzed and the results were concordant in all samples. Approximately 30 percent of the assays were repeated and the results were identical. In addition, 3% of the genotypes were validated by solid-phase minisequencing.

#### Molecular Beacon assay

To analyze the exonic SNPs rs4986934 and rs1801132, 10 ng of genomic DNA was amplified in a 25 μl reaction containing 0.02 U/μl of AmpliTaq Gold DNA polymerase (Applied Biosystems), 3.5 mM MgCl_2_, 250 μM of dNTPs (Amersham Pharmacia Biotech) and 1 μM of PCR primers in TaqMan Buffer A containing the ROX dye (Applied Biosystems). The two MB probes were included at 0.34 μM concentration. The cycling conditions were 95°C for 10 minutes, followed by 40 cycles at 95°C for 30 seconds, 55°C (rs1801132) or 57°C (rs4986934) for 1 minute and 72°C for 45 seconds and was performed using an ABI Prism 7700 Sequence Detection System (Applied Biosystems). The increase in fluorescent signal was registered during the annealing step of the reaction and the end-point signals were used to assign the genotypes as previously described [18]. In each run, positive controls for the three genotypes and negative controls were included. Both possible nucleotides of the SNP were interrogated in the same reaction. Approximately 3% of the assays were repeated and the results were identical. Two percent of the assays were validated by solid-phase minisequencing. The Molecular Beacon assay has previously been quantitatively validated in our laboratory [18].

#### Solid-phase minisequencing assay

Solid-phase minisequencing in microtiter plates [20] was used in part to genotype rs4986934. The assay also served as a reference method for the other SNP assays.

#### Microsatellite assay

The dinucleotide repeat rs2234670 region was amplified using an ABI-877 Integrated Thermal cycler PCR robot with standard reagents (Applied Biosystems) [21]. The cycling conditions were 96°C for 10 minutes followed by 36 cycles of 30 seconds at 96°C, 30 seconds at 57°C, and 1 minute at 72°C with a final extension step for 7 minutes. The HEX-labeled fluorescent PCR products were mixed with an internal-lane standard (GS-500 TAMRA) and separated on a 6% polyacrylamide gel using a 96-well ABI 377 automatic sequenator. Gel data were analyzed using Genescan Analysis 3.1 and allele sizes were determined using Genotyper 2.0 (all Applied Biosystems). In each run, 2–3 negative controls were included. Approximately 1% of the assays were repeated and gave concordant results. After calculation of actual number of TA repeats there was no difference between repeated runs of the same sample. The assay was validated by control sequencing of three different repeat lengths.

### Statistical Methods

We verified that genotype frequencies among controls were in accordance with expectations under Hardy-Weinberg equilibrium (HWE) using χ^2 ^statistics. We considered the rs2234670 in its original form, as 8 categories, or dichotomized, into long and short (≤ 14 or > 14 repeats), as the TA_n _lengths were bimodally distributed with peaks at 11 and 18 repeats, with a dip at 14 repeats. In addition, we estimated pairwise linkage disequilibrium values |D'| and r^2 ^[22,23].

We used conditional logistic regression to calculate odds ratios (OR) and 95 percent confidence intervals (CI) for association between single loci genotypes and endometrial cancer risk. We conditioned on the variables used for selection, i.e. age in 5-year categories, use of menopausal hormones for two years or more, and self-reported diabetes mellitus.

We tested for associations between *ESR1 *polymorphisms and other covariates by scrutinizing 3 × *k *tables and calculating χ^2 ^statistics. Covariates that were approximately normally distributed (body mass index (BMI, weight in kg/height in m^2^) and age at menopause) were tested by means of two-sample t test over genotype categories. The following covariates were considered: age (in 5-year age groups), use of any preparation containing medium potency estrogen with or without added progestin (never, < 2 years, ≥ 2 years), use of preparation containing only medium potency estrogen without progestin (never, < 2 years, ≥ 2 years), BMI (< 25, 25–27.99, ≥ 28 or as a continuous variable), age at menopause (in tertiles among controls or as continuous variable), parity (nulliparous, 1 childbirth, ≥ 2 childbirths), smoking at least 100 cigarettes in their lifetime or regularly for at least 1 year (ever, never), use of combined oral contraceptives (ever, never), age at last birth (tertiles or as a continuous variable, only including women who ever had given birth), and age at menarche (tertiles or as a continuous variable). For detailed descriptions of how the above variables were defined, please see [8]. All covariates were introduced into the logistic regression model to detect any indications of confounding or other associations potentially affecting the primary association between genotypes and endometrial cancer. We used likelihood ratio (LR) tests test to compare successive models, all of which included the same set of endometrial cancer cases and controls. We performed these analyses using the SAS system (Release 8.02, SAS Institute Inc. Cary, NC, USA).

We tested for disease-haplotype association using likelihood-based approaches contained in the software EH plus [24] as well as in routines written in the S-PLUS (Insightful) programming language. Most available software (including EH plus) for testing association between haplotypes and case/control status assume multiplicative penetrance, that is, the OR comparing two haplotype copies against none is assumed to be the square of the OR comparing one against none.

## Results

We successfully genotyped 695 cases and 1564 controls for the rs2234670, 698 cases and 1561 controls for the rs2234693, 698 cases and 1562 controls for the rs9340799, 701 cases and 1563 controls for the rs4986934, and 702 cases and 1563 controls for the rs1801132 marker. In controls, SNP genotype frequencies were in HWE but the rs2234670 genotype frequencies were not, whether in dichotomised form (p = 0.003) or when all alleles were considered (p < 0.0001).

The two intron 1 markers were in strong LD while there was weaker pairwise LD between the remaining markers (Table 1).

**Table 1:** Pairwise linkage disequilibrium between polymorphisms in the estrogen receptor alpha gene

		rs2234670^a^	rs2234693	rs9340799	rs4986934	rs1801132
				**|D'|**		
rs2234670^a^		-	**0.790**	**0.734**	**0.046**	**0.188**
rs2234693		*0.571*	-	**0.998**	**0.825**	**0.338**
rs9340799	r^2^	*0.349*	*0.590*	-	**0.742**	**0.245**
rs4986934		*0.001*	*0.023*	*0.011*	-	**0.822**
rs1801132		*0.008*	*0.029*	*0.009*	*0.090*	-

|D'|-values above diagonal and r^2^-values below.

^a ^For |D'| rs2234670 Ta_n _repeat lengths were divided into eight categories, <10, 11, 12–13, 14–16, 17, 18, 19 or > 20. This ensured that there were few cells that contained less than five observations. For r^2 ^the Ta_n _repeat is dichotomized.

Risk factor exposure differences between cases and controls reflected established associations (Table 2). Ninety-three percent of the tumours in the following analyses were of endometroid histological type. Myometrial invasion was available for 79 percent of the cases, out of which 31 percent had invasion through more than half of the myometrial thickness. Thirty-eight, 45 and 17 percent were of histopathological grade 1, grade 2, and grade 3, respectively. We found no convincing associations between genotypes and any of the covariates (data not shown). There was no difference in genotype frequencies between cases who donated blood samples and those who participated via tissue samples (P = 0.96, 0.66, 0.48, 0.56, and 0.51 for rs2234670, rs2234693, rs9340799, rs4986934, and rs1801132, respectively) or between primary selected controls and controls included after they had been genotyped for the parallel breast cancer study (P = 0.52, 0.98, 0.77, 0.31, 0.29 for rs2234670, rs2234693, rs9340799, rs4986934, and rs1801132, respectively). Further, genotype frequencies did not differ between those included in multivariate models and those excluded due to missing information about one or more of the included covariates (data not shown). Cancers in women participating via tissue samples were more often of more advanced stage as measured by degree of myometrial invasion (no invasion 2.8 vs 10.5 percent, less than half of the myometrial thickness 65.3 vs 61.7 percent, more than half of the myometrial thickness 26.4 vs 25.9 percent, through the serosa 5.6 vs 1.9 percent, p = 0.05).

**Table 2:** Distribution of endometrial cancer risk factors among participating endometrial cancer cases and population controls

Co-variate	Cases/controls	Cases	Controls	
	N	Mean (SD)		P value
Age	702/1563	64.1 (6.80)	63.4 (7.03)	0.0001
Age at menarche	653/1427	13.5 (1.37)	13.5 (1.42)	0.14
Age at last birth	604/1405	29.4 (5.08)	30.4 (5.34)	0.0002
Age at menopause	614/1503	51.0 (4.06)	50.1 (3.91)	<0.0001
Recent BMI	701/1546	27.4 (5.31)	25.5 (4.27)	<0.0001
				
		Percent		
Nulliparous	158/97	14	10	
1 childbirth	289/146	21	18	0.008
2+ childbirths	1116/459	65	71	
				
Family history^a^	667/1397	10	5	<0.0001
Diabetes Mellitus, self-reported^b^	702/1441	10	8	0.17
Ever use of oral contraceptives	699/1550	24	36	<0.0001
Ever use of menopausal hormones^b^	690/1541	28	28	0.97
Smoking^c^	702/1563	35	43	0.0004

^a ^Family history is defined as having a mother or a sister with endometrial cancer. ^ b^Women with self-reported diabetes mellitus and those who had used menopausal hormones containing medium potency estrogens were over-sampled into the study and thus the proportions do not reflect known case-control differences ^c ^Smoking is defined as a participant having smoked at least 100 cigarettes during her lifetime or having smoked regularly for at least one year

Non-participants, i.e. those that were eligible for this study but that either were not selected or did not consent to participate, were on average older than participants. Non-participating cases more often had cancer with more advanced myometrial invasion; 50 percent had cancer invasion to more than half of the myometrial thickness as compared to 31 percent among participants (p < 0.0001). Participants were also more likely to have been oral contraceptives users. Due to the over-sampling scheme, participants were also more likely to have used menopausal hormones. Despite our over-sampling of women with diabetes mellitus, participants were less likely to have this disease than non-participants. Participants and non-participants were similar with regard to smoking habits, family history of endometrial cancer, BMI, age at last birth and age at menopause (data not shown).

### Association with endometrial cancer

In univariate analyses conditioned on the selection variables the rs2234670, the rs2234693 and the rs9340799 were associated with endometrial cancer risk so that subjects homozygous for the rarer alleles (denoted 11 in Table 3) were at decreased risk as compared to homozygotes for the most common allele (denoted 00). None of the covariates appeared to be confounders. However, including information about parity, BMI, smoking status and use of oral contraceptives significantly improved the model fit. Age at last birth and age at menopause further bettered model fit but since adjustment left point estimates unchanged these variables were excluded due to missing information in many individuals. Multivariate analyses yielded slightly stronger results compared to those from univariate analyses (Table 3). The difference could not be attributed to any one variable. Heterozygotes were at intermediate risk. Excluding cases with DNA derived from tissue left estimates largely unaltered and associations using cases with tissue derived DNA did not speak against pooling all successfully genotyped cases in the further analysis (Additional file 1, Supplementary table 3).

**Table 3:** Odds ratios (OR) for endometrial cancer and 95% confidence intervals (CI)

			Marker	rs2234670	rs2234693	rs9340799	rs4986934	rs1801132
Genotype	00^a^	Case/controls		232/441	211/382	324/573	617/1276	406/834
		OR (CI)		1 (ref)	1 (ref)	1 (ref)	1 (ref)	1 (ref)
								
	01^a^	Case/controls		307/618	336/670	280/632	44/89	226/468
		OR (CI)	Univariate^b^	0.95 (0.75–1.19)	0.88 (0.70–1.11)	0.79 (0.64–0.98)	1.02 (0.68–1.54)	1.02 (0.82–1.26)
			Multivariate^c^	0.92 (0.72–1.16)	0.86 (0.68–1.10)	0.75 (0.60–0.93)	1.03 (0.67–1.58)	1.00 (0.80–1.26)
								
	11^a^	Case/controls		123/309	115/316	58/163	1/3	30/66
		OR (CI)	Univariate^b^	0.72 (0.54–0.97)	0.65 (0.48–0.87)	0.59 (0.41–0.85)	0.94 (0.09–9.40)	0.94 (0.58–1.51)
			Multivariate^c^	0.73 (0.54–0.99)	0.65 (0.48–0.89)	0.53 (0.37–0.77)	1.02 (0.09–11.61)	0.95 (0.58–1.57)

Univariate results include individuals with complete information about the covariates used in the multivariate analysis.

^a ^The genotypes are rs2234670: 00 = ≤ 14/≤ 14, 01 = ≤ 14/> 14, 11 => 14/> 14; rs2234693: 00 = T/T, 01 = T/C, 11 = C/C; rs9340799: 00 = A/A, 01 = A/G, 11 = G/G; rs4986934: 00 = C/C, 01 = C/T, 11 = T/T; rs1801132: 00 = C/C, 01 = C/G, 11 = G/G. ^b ^Long-term users of menopausal hormones and diabetics were over-sampled, thus logistic regression models are conditioned for age- and sampling category, which means that only those with complete information about hormone use and diabetes mellitus were included. ^c ^Multivariate models are adjusted for parity (continuous), BMI (continuous), smoking (ever, never) and use of oral contraceptives (ever, never)

We examined whether including more than one marker would improve model fit, indicating influence on risk by combinations of alleles at different loci (i.e. haplotype effects). The association with rs9340799 was the strongest. The addition of either rs2234670 or rs2234693 to a model with rs9340799 did not significantly improve explanation of disease status (LR test p = 0.70 and p = 0.50, respectively). Conversely, starting with rs2234670 or rs2234693 and adding rs9340799 significantly improved model fit (LR test p = 0.02 and p = 0.03, respectively). Adding any of the last two markers, rs4986934 or rs1801132, neither improved fit nor changed estimates nor confidence intervals for the first three markers considered one at the time. When adding to the model with rs9340799 also rs2234693 and the interactions between these loci, the fit was not improved (LR test, p = 0.48). Neither did adding the rs1801132 (LR test p = 0.31) or the rs2234670 (LR test p = 0.83) and the respective interactions improve fit. Only three out of the four possible haplotypes of rs9340799 and rs2234693 existed in our study population (Table 4), so that we were unable to compare the model containing rs9340799 and rs2234693 main effects and interactions with the haplotype model. Other haplotype models (under the assumption of multiplicative penetrance), using rs9340799 and one of rs2234670 or rs1801132, did not provide a significant improvement in fit over the single locus (rs9340799) model.

**Table 4:** Distribution of *ESR1 *four-locus haplotype frequencies as estimated through EM algorithms among endometrial cancer cases and population controls

Locus				Proportion estimated to carry haplotype		
rs2234693	rs9340799	rs4986934	rs1801132	Controls (n = 1560)	All cases (n = 694)	Endometroid cases (n = 647)
T	A	C	C	0.371	0.404	0.406
C	G	C	C	0.288	0.255	0.252
C	A	C	C	0.114	0.118	0.118
T	A	C	G	0.123	0.137	0.135
C	G	C	G	0.055	0.045	0.046
T	A	T	G	0.028	0.025	0.027
						
Proportion accounted for by the most common haplotypes:				0.979	0.984	0.984

We investigated the association between rs9340799 and endometrial cancer stratified by the covariates (Table 5). We were primarily interested in potential interactions between the marker and use of menopausal hormones and BMI, since these factors are the most important determinants of serum estrogen levels in postmenopausal women, though we also examined other hormonally related variables. We could not estimate main effects of variables that we used in our sampling scheme (see Methods section) and thus the results presented (first in Table 5) from the analyses stratified by these factors convey only genetic effects. In subdividing the cases and controls by use of menopausal hormones, the protective effect of rs9340799 emerged among never users while the pattern was unclear among users, possibly due too small numbers of observations (Table 5). P-values for interaction were 0.007, 0.35, and 0.08 for compounds containing estrogen only, estrogen plus progestins cyclically and estrogen plus progestins continuously, respectively. Despite the small p-values, there were no allele-dose response relations. Association analyses stratified by various other risk factors for endometrial cancer are given in Table 6. BMI did modify the association between rs9340799 and endometrial cancer (p = 0.91). This result was not sensitive to BMI category cutpoints (p = 0.75 and p = 0.45 using BMI>30 or BMI>31 as the highest category, respectively). The protective effect of rs9340799 was clearer among non-smokers and among non-users of combined oral contraceptives although tests for interaction were non-significant (p = 0.17 and p = 0.19, respectively). There appeared to be no modification of the association between rs9340799 and endometrial cancer risk by use of oral low potency estrogen (p = 0.50), diabetes mellitus (p = 0.91), parity (p = 0.88), age at menopause (in tertiles, p = 0.72), age at last birth (in tertiles, p = 0.46), or family history of endometrial cancer (p = 0.51).

**Table 5:** Odds ratios (OR) and 95% confidence intervals (CI) estimating the association between *ESR1 *rs9340799 and endometrial cancer by subgroups of hormone use and self-reported diabetes mellitus

		ORs and CIs for rs9340799 genotypes^a^		
		
Covariate	Cases/controls	A/A	A/G	GG
Use of menopausal hormones with medium potency estrogens				
Never users	485/1006	1 (ref)	0.78 (0.62–0.97)	0.51 (0.34–0.77)
				
Ever use of estrogen only	85/104	1 (ref)	0.47 (0.23–0.97)	1.96 (1.62–6.14)
Ever use of estrogen plus progestins cyclically	100/196	1 (ref)	0.85 (0.49–1.49)	0.78 (0.33–1.84)
Ever use of estrogen plus progestins continuously	38/145	1 (ref)	0.57 (0.24–1.35) p = 0.08^b^	0.94 (0.29–3.08)
				
Diabetes Mellitus	598/1258	1 (ref)	0.76	0.56
No			(0.61–0.94)	(0.39–0.81)
Yes	64/110	1 (ref)	0.52 (0.25–1.09) p = 0.91^b^	0.57 (0.15–2.15)

^a ^Analyses were conditioned on age, hormone use for more than two years, and diabetes mellitus ^b ^Test for interactions with variables used for selection were designed to test if the terms for the two (two level covariate) or four (three level covariate) extra combined categories were different from zero in a model containing the main effect of genotype and covariate. For medium potency estrogen variables the test was carried out comparing ever use with never use of any medium potency estrogens.

**Table 6:** Odds ratios (OR) and 95% confidence intervals (CI) estimating the association between *ESR1 *rs9340799 and endometrial cancer by subgroups of other endometrial cancer risk factors

		ORs and CIs for rs9340799 genotype^a^			
Covariate	Case/contl	A/A	A/G	GG	P for interaction
Use of oral low potency estrogen (estriol)					
Never	533/1213	1 (ref)	0.79 (0.64–0.99)	0.55 (0.37–0.81)	
Ever	127/153	1.77 (1.20–2.61)	1.28 (0.86–1.92)	1.52 (0.74–3.15)	0.50
					
BMI (kg/m^2^)					
< 25	265/698	1 (ref)	0.83 (0.61–1.13)	0.62 (0.37–1.03)	
25–28	136/387	1.01 (0.71–1.45)	0.70 (0.48–1.02)	0.59 (0.27–1.27)	
> 28	261/283	2.90 (2.05–4.11)	2.05 (1.46–2.87)	1.39 (0.80–2.43)	p = 0.93
					
Smoking^b^					
Never	434/783	1 (ref)	0.84 (0.05–1.08)	0.51 (0.33–0.80)	
Ever	228/585	0.75 (0.56–1.00)	0.50 (0.37–0.68)	0.59 (0.35–0.98)	p = 0.17
					
Use of combined oral contraceptives					
Never	500/863	1 (ref)	0.86 (0.68–1.09)	0.58 (0.38–0.87)	
Ever	162/505	0.63 (0.46–0.87)	0.38 (0.27–0.54)	0.43 (0.24–0.78)	p = 0.19
					
Parity					
0 childbirths	94/137	1 (ref)	0.78 (0.44–1.38)	0.44 (0.16–1.24)	
1 childbirth	135/251	0.69 (0.41–1.18)	0.62 (0.37–1.04)	0.51 (0.23–1.11)	
≥ 2 childbirths	433/980	0.62 (0.39–0.96)	0.45 (0.29–0.70)	0.37 (0.21–0.65)	p = 0.88
					
At at menopause (years)					
< 50	157/447	1 (ref)	0.81 (0.53–1.20)	0.51 (0.25–1.05)	
50–51.5	125/393	0.98 (0.64–1.48)	0.68 (0.45–1.04)	0.79 (0.40–1.55)	
> 51.5	294/470	1.75 (1.23–2.51)	1.48 (1.03–2.12)	0.95 (0.54–1.66)	p = 0.72
					
Age at last birth (years, among parous only)					
≤ 27	209/364	1 (ref)	0.66 (0.46–0.97)	0.75 (0.40–1.41)	
27.5–32.5	194/452	0.75 (0.52–1.09)	0.53 (0.36–0.79)	0.33 (0.17–0.64)	
≥ 33	165/415	0.55 (0.37–0.81)	0.52 (0.35–0.77)	0.39 (0.20–0.75)	p = 0.46
					
Family history of endometrial cancer^c^					
No	564/1262	1 (ref)	0.78 (0.63–0.97)	0.63 (0.44–0.90)	
Yes	65/67	2.56 (1.47–4.46)	1.28 (0.76–2.18)	1.48 (0.39–5.66)	p = 0.51

^a ^Analyses were conditioned on age, hormone use for more than two years, and diabetes mellitus ^b ^Smoking is defined as having smoked at least 100 cigarettes or having smoked regularly for at least one year ^c ^Family history is defined as having a mother or a sister with endometrial cancer.

Only 45 cases were of non-endometroid histotype. Excluding these from the analyses did not change the results of any of the above analyses (data not shown).

Analyses where cases were stratified by degree of myometrial invasion (Additional file 1, Supplementary table 3) indicated a more pronounced protective effect of the rs9340799 variant allele among those with invasion through more than 50 percent of the myometrium (adjusted OR 0.67 CI 0.45–0.98 for heterozygotes and OR 0.35 CI 0.16–0.75 for variant allele homozygotes). This latter analysis was based on only 139 cases. Stratifying on histopathological grade did not reveal any clear pattern to indicate a differential effect of genotype on risk for endometrial cancers of various degree of differentiation (Additional file 1, Supplementary table 4).

## Discussion

We show that non-coding variation in *ESR1 *is associated with endometrial cancer risk. With regard to the rs9340799 locus, homozygotes for the rare G allele had an almost halved risk for endometrial cancer compared to common allele homozygotes. This protection seemed more pronounced when only cases with invasion through more than 50 percent of the myometrium were considered. The latter analysis was based on few cases and needs further confirmation. We found no convincing modification of this association by other endometrial cancer risk factors or histopathological grade.

We found no evidence of additional effects of other markers than rs9340799. However, we cannot determine whether this SNP is functional or in LD with some other functional locus. Furthermore, we cannot be completely certain that rs9340799 is more influential than rs2234693 because of the strong LD between these markers, separated by only 46 bp.

The association between rs9340799 and endometrial cancer was not convincingly modified by exposures that alter the availability of estradiol or estrone such as use of menopausal hormone preparations or high BMI. In our parallel breast cancer study [25] the *ESR1*-disease risk association was stronger in women with high BMI but this appeared not to be the case in endometrial cancer. The effect of rs9340799 was largely the same in any stratum with sufficient sample size.

Our study is population-based and the largest investigating the relation between *ESR1 *and endometrial cancer to date. Furthermore, the study population is genetically homogenous, which limits concern about population stratification and which also preserves power as no additional stratifications based on ethnicity need to be made. The vast majority of cases in this study were of the strongly estrogen related endometroid type, and thus constitute a sample in which functional *ESR1 *alterations are expected to become evident.

The markers that we have studied here were selected because they were known to be polymorphic according to the literature available when the study was planned (around 1996). They were not selected, as is usually the case with current studies, in an attempt to represent the entire gene. This is a weakness but it should not discredit the reported association.

It appears as if the *ESR1 *gene is composed of three different LD blocks [26]. All markers that are typed in the present study are located within the first region, towards the 5' end of the gene. We did not find evidence of any interactions between markers, which would have indicated that functional combinations of alleles exist in the gene. We initially intended to investigate if we could establish whether any SNP was functional or instead in LD with a functional locus, by comparing a model with genotype main effects and interactions, to a haplotype model, using SNPs rs9340799 and rs2234693. If the haplotype model had exhibited a significantly better fit then there would have been indication that other, linked, un-typed, SNPs were responsible for the association [27]. However, only three out of the four possible haplotypes of rs9340799 and rs2234693 exist in our data, so that we could not make this comparison. In contrast to our findings in the parallel breast cancer study [25], a haplotype model with rs9340799 and rs1801132 did not provide any additional explanatory information.

Our main results replicate our own findings in a separate but equivalent Swedish study population where we observed associations of similar strength and magnitude [6]. They also corroborate results of a small Japanese study where rs9340799 GG and rs2234693 CC genotypes were associated to a lower risk for endometrial cancer compared to those homozygous for the common allele [4]; OR 0.26 (CI 0.09–0.79) and OR 0.23 (CI 0.07–0.82), respectively. Another Japanese study found no such association [5] but a decreased risk with allele-dose effect with the rare allele of a *ESR1 *codon 10 SNP.

Weel and colleagues found the rs2234693 CC genotype to be associated with an earlier onset of natural menopause [28], which would entail a lower risk of endometrial cancer, but we did not find any similar association, nor any interaction between *ESR1 *genotype and age at menopause.

There is no evidence in the literature that rs9340799 affects the amount or function of the estrogen receptor protein. Herrington and colleagues found that the adjacent rs2234693 C allele produced an additional binding site for the myb family of transcription factors [29]. Binding of B-myb to this site appeared to have the capability of enhancing *ESR1 *transcription. Since B-myb is in itself estrogen responsive there is a possibility of a positive feedback loop that could amplify estrogenic response. It is possible that in fact rs2234693 is the functional variant and that our finding was due to the strong LD between the markers in addition to random variation in the data. Ongoing studies that include dense mapping of *ESR1 *variants will give guidance as to what variants need to be further functionally validated.

Although our results, which are in line with a priori theories, might indicate a true influence of *ESR1 *variation on estrogen dependent phenotypes they could conceivably be due to selection bias, a potential problem in case-control studies. Our participation rates, 88 and 76 percent among cases and controls, respectively (67 and 64 percent using those eligible for the parent study in the denominator) could lead to bias if participation were related to genotype. Non-participating cases had a more advanced disease as measured by myometrial invasion. If, for example, a genetic variant is associated to severe cancer but not to less severe cancer and the more severe cases are less likely to participate because they have died, any association with cancer overall would be biased towards the null. Furthermore, genotype frequencies were not different between participants who donated blood and those who took part via a tissue sample, the latter of which were more often deceased. Our results cannot be generalized to include all endometrial cancer because there is data that indicates differing etiologies between endometroid and non-endometroid endometrial cancer [1].

Another possible mechanism for selection bias is if an *ESR1 *variant influences a phenotype that differentially affects ascertainment or recruitment probability among cases and controls. In this case one would expect to find case-control associations with multiple otherwise unrelated phenotypes. *ESR1 *variation has indeed been associated with a host of other outcomes such as coronary heart disease [30], height [31], bone mineral density [32,33], multiple sclerosis [34], cognitive impairment [35], and age at menarche [36]. However, despite the diversity among these outcomes there is reasonable evidence of etiological roles for estrogen. Our comparisons of characteristics among participants and non-participants do not point to any plausible mechanism for selection bias as an explanation to the observed association.

## Conclusion

In conclusion, we found an association between genetic variation in *ESR1 *and endometrial cancer risk; the rs9340799 GG genotype was associated to an almost 50 percent decreased risk for endometrial cancer compared to the AA genotype. Our results strengthen belief in this *a priori *strong candidate gene but we cannot, based on our data, establish the importance of any single genetic locus within the gene.

## Competing interests

The authors declare that they have no competing interests.

## Authors' contributions

SW planned and coordinated the collecting of biological samples, participated in the genotyping, performed most of the biostatistical analyses, interpreted results, and wrote the manuscript. LL performed the bulk of the genotyping and took part in interpreting results and writing the manuscript. KH oversaw all and performed some of the biostatistical analyses, interpreted the results. CM, IP, JB and EW planned the study and interpreted the results. HM, ACS, AK, UL, MLF planned, coordinated and oversaw all DNA extraction and genotyping. FS performed genotyping. All authors critically read and took part in finalizing the manuscript.

## Pre-publication history

The pre-publication history for this paper can be accessed here:



## Supplementary Material

Additional file 1 **Supplementary material Wedren ESR1 endometrial cancer.** Tables of primers and probes used for genotyping and tables of main results stratified by DNA source, myometrial invasion, and histological grade. Click here for file
