# Supplementary material for: Estrogen receptor alpha gene polymorphism and endometrial cancer risk – a case-control study
Source: BMC Cancer. 2008 Nov 6;8:322. doi: 10.1186/1471-2407-8-322 (PMC2613912; doi:10.1186/1471-2407-8-322)
Supplement: Additional file 1 — Supplementary material Wedren ESR1 endometrial cancer. Tables of primers and probes used for genotyping and tables of main results stratified by DNA source, myometrial invasion, and histological grade. [file 1471-2407-8-322-S1.pdf]

Supplementary material

Wedrén et al – Estrogen receptor alpha gene polymorphism and endometrial cancer risk- a case-control study

Supplementary table 1 PCR primers used to genotype the estrogen receptor alpha gene

	Sequence 5’ -> 3’ and modifications	
Polymorphism	Forward	Reverse
Promotor Rs2234670 [a]	*HEX* - GAT TAT AGA CGC ATG ATA TAC TTC ACC	GGA TAT GCA GAA TCA AAT ATC CAG ATG
Intron 1 rs2234693 and rs9340799 [b]	(*Bio* -) GTG TTG TCC ATC AGT TCA TCT	(*Bio* -) AGA ACC ATT AGA GAC CAA TGC
Exon 3 rs4986934 (MB) [c]	CCA ACC AGT GCA CCA TTG ATA	CCT TTC ATC ATT CCC ACT TCG
Exon 3 rs4986934 [d]	AAC AGG AGG AAG AGC TGC CAG GC	*Bio* - CTT TCA TCA TTC CCA CTT CGT AGC A
Exon 4 rs1801132 (MB) [c]	CAG ATG GTC AGT GCC TTG TTG GA	CGA AGC TTC ACT GAA GGG TCT GG
Exon 4 rs1801132 [d]	TGA CGG CCG ACC AGA TGG TCA GT	*Bio* - AGG GTC TGG TAG GAT CAT ACT CGG

[a] Forward primer labeled with 6-carboxyhexachlorofluorescein (HEX) to enable detection on the sequencing instrument.[b] Primers used for minisequencing with fluorescence polarization detection. For validation by solid-phase minisequencing one of the PCR primers was substituted by a biotinylated (Bio) primer with the same sequence to enable solid-phase capture on streptavidin coated microtiter plates. [c] Primers optimized for the molecular beacon assay (MB) [d] For validation by solid-phase minisequencing we used a second pair of PCR primers, of which the reverse primer was biotinylated (Bio) to enable solid-phase capture on streptavidin coated microtiter plates.

Supplementary table 2 Primers and probes used for genotyping the estrogen receptor alpha gene

Assay	Polymorphism		Sequence 5’ -> 3’ and modification
Minisequencing	Intron 1 rs2234693 [a]	Non-coding	TGG GAA ACA GAG ACA AAG CAT AAA AC
		Coding	CAT CTG AGT TCC AAA TGT CCC AGC
Minisequencing	Intron 1 rs9340799 [a]	Non-coding	GAC CAA TGC TCA TCC CAA CTC
		Coding	TCC CAG AGA CCC TGA GTG TGG TCT
Molecular Beacon	Exon 3 rs4986934 [b]		FAM - ccg agc CGG CTC CG**C** AAA TGC TAg ctc gg - DABCYL
			TET - ccg agc CGG CTC CG**T** AAA TGC TAg ctc gg - DABCYL
Molecular Beacon	Exon 4 rs1801132 [b]		FAM - cca agc GAG CCC CC**C** ATA CTC TAg ctt gg - DABCYL
			TET - cca agc GAG CCC CC**G** ATA CTC TAg ctt gg – DABCYL
Minisequencing	Exon 3 rs4986934 [c]		TGC CAG GCC TGC CGG CTC CG
Minisequencing	Exon 4 rs1801132 [c]		TTG TTG GAT GCT GAG CCC CC

[a] Primers used for minisequencing with fluorescence polarization detection in both directions and for validation by solid-phase minisequencing. [b] Two molecular beacon probes, each corresponding to one of the variable nucleotides and labeled with 5-carboxyfluorescein (FAM) or 6-carboxytetrachlorofluorescein (TET) and dimethylaminophenylazobenzoic acid (DABCYL) were used in the same reaction in the molecular beacon assay. Small letters indicate the stem part and bold letters indicate the nucleotide complementary to the SNP position [c] Primers used for validation by solid-phase minisequencing.

Supplementary table 3 Odds ratios for endometrial cancer associated with various genotypes of the *ESR1* rs9340799 and 95% confidence intervals from logistic regression models using homozygous for the most common allele (AA) as the reference, stratified by DNA source.

	Unadjusted			Adjusted*		
	A/A	A/G	G/G	A/A	A/G	G/G
DNA source						
Blood	1 (ref)	0.76 (0.61-0.96)	0.63 (0.44-0.92)	1 (ref)	0.74 (0.59-0.94)	0.58 (0.39-0.85)
Tissue	1 (ref)	0.98 (0.62-1.57)	0.35 (0.12-1.01)	1 (ref)	0.88 (0.54-1.43)	0.29 (0.1-0.88)

* Adjusted for parity (continuous), BMI (continuous), smoking (ever, never) and use of oral contraceptives (ever, never)

Supplementary table 4 Odds ratios associated with various genotypes of the *ESR1* rs9340799 and 95% confidence intervals from logistic regression models with homozygous for the most common allele (AA) as the reference group, stratified by myometrial invasion and grade.

	Unadjusted			Adjusted*		
	A/A	A/G	G/G	A/A	A/G	G/G
Myometrial invasion						
<50%	1 (ref)	0.92 (0.71-1.21)	0.86 (0.56-1.32)	1 (ref)	0.87 (0.65-1.14)	0.79 (0.51-1.22)
>50%	1 (ref)	0.69 (0.47-1.00)	0.38 (0.18-0.82)	1 (ref)	0.67 (0.45-0.98)	0.35 (0.16-0.75)
Grade						
Grade 1	1 (ref)	0.73 (0.52-1.02)	0.92 (0.56-1.49)	1 (ref)	0.68 (0.49-0.96)	0.84 (0.51-1.38)
Grade 2	1 (ref)	0.78 (0.58-1.04)	0.33 (0.17-0.63)	1 (ref)	0.73 (0.54-0.99)	0.29 (0.15-0.56)
Grade 3	1 (ref)	0.84(0.54-1.31)	0.58 (0.26-1.31)	1 (ref)	0.85 (0.54-1.34)	0.46 (0.2-1.09)

* Adjusted for parity (continuous), BMI (continuous), smoking (ever, never) and use of oral contraceptives (ever, never)
